# A Unique Combination of Mn^2+^ and Aluminum Adjuvant Acted the Synergistic Effect

**DOI:** 10.1155/2024/7502110

**Published:** 2024-04-17

**Authors:** Yuwei Cen, Shujie Chen, Shuyu Wei, Shuangshuang Wu, Mingyang Tao, Youxi Fu, Yuncheng Wang, Jing Chen, Yixuan Ma, Hongyan Liu, Baifen Song, Jinzhu Ma, Beiyan Wang, Yudong Cui

**Affiliations:** ^1^College of Life Science and Technology, Heilongjiang Bayi Agricultural University, Daqing 163319, China; ^2^Key Laboratory of Animal Epidemiology and Zoonosis, College of Veterinary Medicine, China Agricultural University, Beijing 100083, China

## Abstract

**Introduction:**

The development of combinatorial adjuvants is a promising strategy to boost vaccination efficiency. Accumulating evidence indicates that manganese exerts strong immunocompetence and will become an enormous potential adjuvant. Here, we described a novel combination of Mn^2+^ plus aluminum hydroxide (AH) adjuvant that significantly exhibited the synergistic immune effect. *Methodology*. Initially, IsdB3 proteins as the immune-dominant fragment of IsdB proteins derived from *Staphylococcus aureus* (*S. aureus*) were prepared. IsdB3 proteins were identified by western blotting. Furthermore, we immunized C57/B6 mice with IsdB3 proteins plus Mn^2+^ and AH adjuvant. After the second immunization, the proliferation of lymphocytes was measured by the cell counting kit-8 (CCK-8) and the level of IFN-*γ*, IL-4, IL-10, and IL-17 cytokine from spleen lymphocytes in mice and generation of the antibodies against IsdB3 in serum was detected with ELISA, and the protective immune response was assessed through *S. aureus* challenge.

**Results:**

IsdB3 proteins plus Mn^2+^ and AH obviously stimulated the proliferation of spleen lymphocytes and increased the secretion of IFN-*γ*, IL-4, IL-10, and IL-17 cytokine in mice, markedly enhanced the generation of the antibodies against IsdB3 in serum, observably decreased bacterial load in organs, and greatly improved the survival rate of mice.

**Conclusion:**

These data showed that the combination of Mn^2+^ and AH significantly acted a synergistic effect, reinforced the immunogenicity of IsdB3, and offered a new strategy to increase vaccine efficiency.

## 1. Introduction

Adjuvant is able to increase the production of various cytokines, antigen processing and presentation about antigen presenting cells to boost humoral and cellular immune responses triggered by antigens; therefore, it is very critical for strengthening immunogenicity of vaccine [1–4]. A lot of adjuvants, including oil emulsion, aluminum adjuvant, and nanosphere adjuvant, act to retain antigens and slow the release of antigens, therefore, the adjuvant is able to assist vaccines in enhancing the magnitude and durability of antigen-specific immune responses [5, 6]. However, a single adjuvant often elicits weak activity and does not achieve the desired immune effect. Therefore, the development of multiple or combinatorial adjuvants can induce a broader humoral immune response and desired cell-mediated immune response, which is a promising approach for increasing the effect of vaccines [7, 8].

Manganese (Mn) is an important trace element in the tissues of mammals, ranging from 0.3 to 2.9 mg/kg at tissue wet weight, and performs different physiological processes, mainly including immunomodulation, antioxidant defense, development, reproduction, and neuronal function [9–12]. Importantly, Mn^2+^ plays an important role in promoting STING's activity by enhancing the affinity of cGAMP-STING and improving the antiviral and antitumor immune responses of the body. Mn-deficient mice decreased cytokine production and were more vulnerable to DNA viruses, indicating that Mn is required for the host defense against DNA viruses. In addition, Mn^2+^ exerts the function of immunologic adjuvant, for instance, colloidal manganese salt increases the efficacy of rabies vaccines in mice, cats, and dogs [13, 14].

Aluminum adjuvant, the unique adjuvant approved by the U. S. Food and Drug Administration (FDA), and the most used adjuvant in licensed human vaccines, may divert antigens from their typical trafficking route toward endolysosomal compartments by its adsorption, which can sharply augment antigen persistency, antigen uptake, and local immunostimulatory response [15, 16]. The aluminum adjuvant is able to adsorb antigens in solution and generates immunogenic particles, and it triggers a Th2-biased immune response by activating the NALP3 inflammasome, followed by the secretion of IL-1*β*, IL-18, and IL-33 [15, 17–19]; however, poor Th1-type immune response induced by aluminum adjuvant makes it inappropriate as a single component in some vaccines.

Recently, the combinatorial adjuvants have been well-studied in clinical practice. For example, aluminum hydroxide (AH) plus cytosine preceding guanosine (CpG) adjuvant triggered high levels of SARS-CoV-2-spike-specific IgG antibodies and neutralized antibodies against SARS-CoV-2 in rats [20], and combination adjuvant of aluminum hydroxide with poly (I : C) and CpG induced a stronger IgG1 and IgG2a immune response and boosted antigen uptake and activation of dendritic cells *in vitro*, by triggering an inflammatory response at the injection site similar to AH [21]. In addition, the formulation of cationic alpha-D-glucan nanoparticles and poly (I : C) adjuvant enhanced various cytokine mRNA expression in lymph nodes, induced high levels of virus-neutralizing antibodies in bronchoalveolar lavage fluid, and reduced microscopic lung lesions and virus load after swine influenza virus challenge [22]. Consequently, the combinatorial adjuvants have exhibited great potential for improving the immunogenicity of vaccines.

*Staphylococcus aureus* (*S. aureus*) seriously threatens human and animal health, however, up to now, an effective vaccine against *S. aureus* is not yet available on the market [23, 24]. IsdB3, as the immunodominant domain of the iron surface determinant B protein (IsdB) from *S. aureus* shows strong immunogenicity, but its immunoprotective effect is yet to be further enhanced, which indicates a new adjuvant to boost the immunogenicity of IsdB3 proteins is very imperative for preventing *S. aureus* infection.

Therefore, we predicted that the combination of Mn^2+^ and aluminum adjuvant could act as a synergistic function to strengthen the immune response triggered by IsdB3. Here, we prepared the immunogens involved in Mn^2+^ and AH plus IsdB3 proteins to evaluate the effect of the novel combined adjuvants.

## 2. Materials and Methods

### 2.1. Reagents, Bacterial Strains, and Mice

ELISA kits for FN-*γ*, IL-4, IL-10, and IL-17 of mice were obtained from Dakewe Biotech Co., Ltd (Beijing, China). Isopropyl-*β*-D−1-thiogalactopyranoside (IPTG) was ordered from Biosharp Company (Hefei, China). Cell counting kit-8 (CCK-8) and BCA protein quantification kits were ordered from Solarbio Company (Shanghai, China). *Staphylococcus aureus* (*S. aureus*) strain Newman and *Escherichia coli* strain BL21 with recombinant pET-32a (+)—*Isdb3* plasmids were provided by the laboratory of Cell Biology, Heilongjiang Bayi Agricultural University. Female C57/B6 mice (20–22 g), 6 to 8 weeks old, were purchased from Yisi Experimental Animal Technology Co., Ltd (Changchun, China).

### 2.2. Expression and Purification of Antigenic Proteins

IsdB3 antigenic proteins, as the immune-dominant fragment of IsdB proteins derived from *S. aureus*, were expressed by pET-32a (+)—*Isdb3 Escherichia coli* strains BL21 with the inductive agent of 0.1 mM IPTG at 37°C for 4 h, then, the bacterial cells were ultrasonicated, and the supernatant was collected. IsdB3 proteins with his His-tags were purified by using His-Binding-resin (Novagen, Germany) according to the manufacturer's instructions, and IsdB3 proteins were confirmed by using SDS-PAGE and the western blot method [25].

### 2.3. Mice Immunization

After IsdB3 proteins were obtained, we prepared the different immunogens, mainly including IsdB3 + Mn^2+^ (manganese chloride (MnCl_2_)) (Sigma-Aldrich, St. Louis, MO), IsdB3 + aluminum hydroxide (AH) (Sigma-Aldrich, St. Louis, MO), and IsdB3 + Mn^2+^ + AH adjuvant. C57/B6 mice were randomly divided into five groups, including IsdB3, IsdB3 + Mn^2+^, IsdB3 + AH adjuvant, IsdB3 + Mn^2+^ + AH adjuvant, and phosphate buffer solution (PBS) + Mn^2+^ + AH adjuvant group (PBS group), and there were 20 mice in each group. C57/B6 mice were inoculated intramuscularly with the dosage of 100 *μ*g of IsdB3 proteins alone or with Mn^2+^ of 140 *μ*g or AH (3%) or Mn^2+^ + AH adjuvant on 0 day and 14 days, respectively. All the animals were fed in a special pathogen‐free environment.

### 2.4. Detection of Cytokine Level

The amount of cytokine was analyzed with an enzyme‐linked immunosorbent assay (ELISA). In brief, seven days after the last immunization, lymphocytes were separated from spleens of mice with lymphocyte separation fluid, then, spleen lymphocytes (1 × 10^6^ cells/ml) were cultured into 96‐well culture plates with 1640 medium supplemented with 10% fetal bovine serum alone as a negative control or with phorbol myristate acetate (PMA, 50 ng/ml) as a positive control or with the desired proteins (IsdB3, 10 *µ*g/ml) at 37°C for 48 h, and then, the cell supernatants were collected and analyzed by ELISA kits for IFN-*γ*, IL-4, IL-10, and IL-17 according to the manufacturer's instructions.

### 2.5. Spleen Lymphocyte Proliferation

The experiment of lymphocyte proliferation was performed as previously described [25, 26]. In brief, seven days after the last immunization, the spleen lymphocytes from the immunized mice were prepared, and then, 100 *μ*L cell suspension (1 × 10^6^ cells/mL) was added into the desired well of a 96-well plate. The IsdB_3_ proteins were used as stimulants at the concentration of 10 *μ*g/ml, with ConA as a positive control and PBS as a negative control. After 48 h, a cell counting kit-8 (CCK-8) was used to detect the cell proliferation response. The sample absorbance at OD_450nm_ was estimated with a microplate reader (Bio-Rad, Hercules, CA, USA), and the stimulation index (SI value) was determined on the basis of OD_450nm_.

### 2.6. Bacterial Load Assay

The bacterial load assay was carried out as previously described [27, 28]. In brief, fourteen days after the last immunization, *S. aureus* strain Newman was used to inject intraperitoneally and infect the immunized mice. After 48 h, the organs including livers, spleens, and kidneys of mice were obtained under aseptic conditions, then the grinding fluids of these organs were prepared and diluted by tenfold gradients, and these diluents were cultured on the tryptic soy agar (TSA) solid plate at 37°C for 12 h. Finally, the bacterial load of these organs was determined according to the colonies on the plates.

### 2.7. Detection of Specific Antibodies

The serum in peripheral blood from immunized mice was separated at 7 days after the last immunization, and then, the level of IgG against IsdB3 was evaluated by indirect ELISA. In brief, IsdB3 proteins were incubated on the 96-well plate as a coating antigen at the concentration of 10 *μ*g/mL for overnight at 4°C. After washing three times with PBS-T, the solution of 3% BSA was added into the wells and blocked for 2 h at 4°C. After washing, the sera of a twofold serial dilution were used as the primary antibody and incubated for 2 h at 37°C. Then, HRP-conjugated goat antimouse IgG mAbs (Promega, San Luis Obispo, CA, USA) as antibodies of detection were added into wells, 2 h after the incubation at 37°C, the plates were incubated with 3, 3′, 5, 5′‐tetramethylbenzidine (TMB) solution for 15 min, and then, 2 N sulfuric acid was used to stop the reaction [29]. Finally, the OD_450nm_ (optical densities) value was detected with an ELISA plate reader.

### 2.8. Opsonophagocytosis Assay

Opsonophagocytosis assay was carried out according to [27, 28]. In brief, wild-type (WT) mice were treated intraperitoneally with 1 mL (60 mg/mL) of thioglycolate solution for 72 h, and then, the peritoneal macrophages were collected and cultured in 6-well plates, and after 24 h, 30 *μ*L of serum in immunized mice was incubated with 1.5 × 10^6^ CFU of *S. aureus* strain Newman at 37°C for 2 h. Then, the mixture was used to treat the macrophages, and after 4 h, these macrophages were incubated with gentamicin of 100 *μ*g/mL to kill the extracellular bacteria outside the macrophages, and after 1 h, the lysates of macrophages were obtained and cultured on TSA plates at 37°C for 12 h. Finally, the number of phagocytized bacteria was confirmed.

### 2.9. Mice Challenge

Fourteen days after the last immunization, the immunized mice were intraperitoneally challenged with a lethal dose of 1.5 × 10^9^ CFU of *S. aureus* strain Newman. The mice were observed and recorded every day after infection. Finally, the survival rate of the challenged groups was determined within 14 days.

### 2.10. Statistical Analysis

The experimental data were analyzed with a one-way ANOVA test by using SPASS software, and the final data are presented as mean ± SD (^*∗*^*P* < 0.05, ^*∗∗*^*P* < 0.01, and ^*∗∗∗*^*P* < 0.001).

## 3. Results

### 3.1. Preparation of Antigenic Proteins

**Figure 1:**
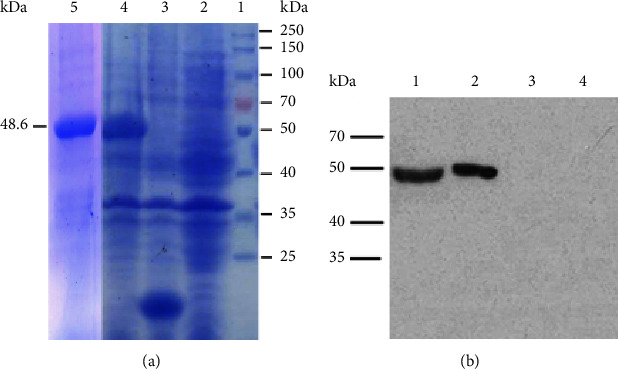
Identification of IsdB3 proteins. (a) Confirmation of IsdB3 proteins by using SDS-PAGE. Lane 1: protein marker; lane 2 and 3: *E. coli* BL21 and *E. coli* BL21 with pET-32a (+) induced by IPTG as control, respectively; lane 4: *E. coli* BL21 with pET-32a (+)—*Isdb3* induced by IPTG; and lane 5: the purification of IsdB3 proteins. (b) The IsdB3 proteins were detected with western blotting. Lane 1: the purification of IsdB3 proteins; lane 2: *E. coli* BL21 with pET-32a (+)—*Isdb3*; lane 3: *E. coli* BL21 with pET-32a (+); and lane 4: *E. coli* BL21.

After the recombinant *Escherichia coli* strain BL21 containing the recombinant pET-32a (+)—*Isdb*3 plasmids were recovered, these bacteria were treated to express IsdB_3_ proteins with IPTG inducer, and then, the His-tagged IsdB3 proteins were purified with His-Bind resin. Finally, SDS-PAGE results revealed that 48.6 kDa fusion proteins were expressed and subsequently purified (Figure 1(a)), and western blotting detection also showed that IsdB3 proteins were correctly expressed and acquired (Figure 1(b)).

### 3.2. Detection of Cytokine

**Figure 2:**
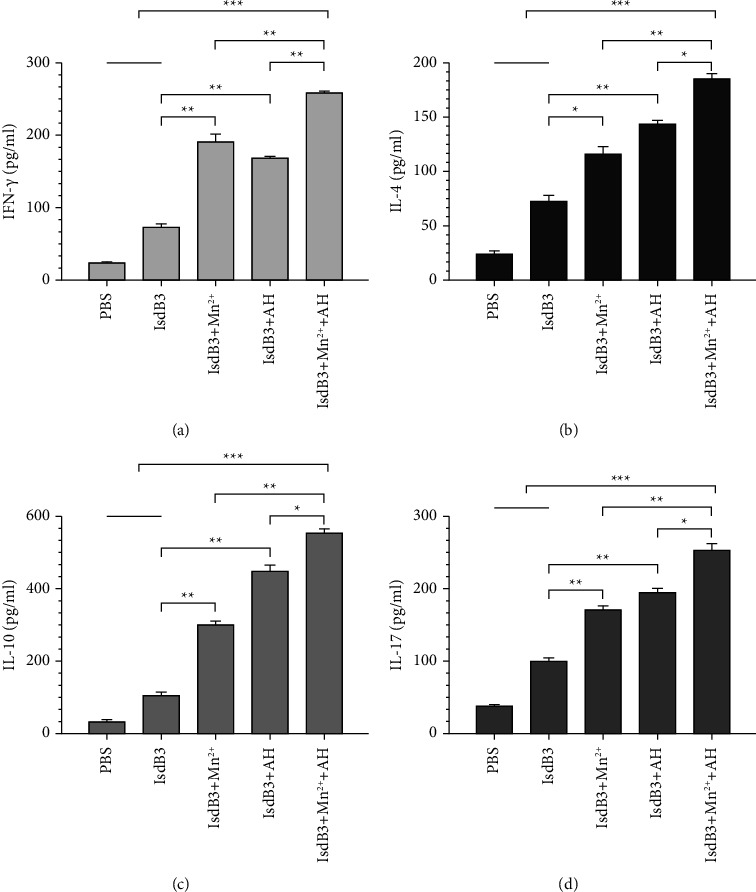
Evaluation of cytokine from splenocytes. Seven days after the last immunization, splenocytes from each group were stimulated with IsdB3 proteins, and the levels of IFN-*γ*, IL-4, IL-10, and IL-17 in the supernatant were detected by the ELISA method. The splenocyte response for IFN-*γ* (a), IL-4 (b), IL-10 (c), and IL-17 (d). The statistical differences between the groups are shown as ^*∗*^*P* < 0.05, ^*∗∗*^*P* < 0.01, and ^*∗∗∗*^*P* < 0.001. The data are shown as the mean ± SD (*n* = 3).

The detection of cytokine levels is shown in Figure 2. The levels of IFN-*γ*, IL-4, IL-10, and IL-17 from the IsdB3 + Mn^2+^ + AH group were the highest among all immune groups. In addition, the levels of IFN-*γ*, IL-4, IL-10, and IL-17 from the IsdB3 + AH group and the IsdB3 + Mn^2+^ group were significantly higher than that of the IsdB3 group and the PBS group, indicating that the combination of Mn^2+^ and AH adjuvant was able to enhance the activation of Th1, Th2, and Th17 cells.

### 3.3. Results of Spleen Lymphocyte Proliferation

**Figure 3:**
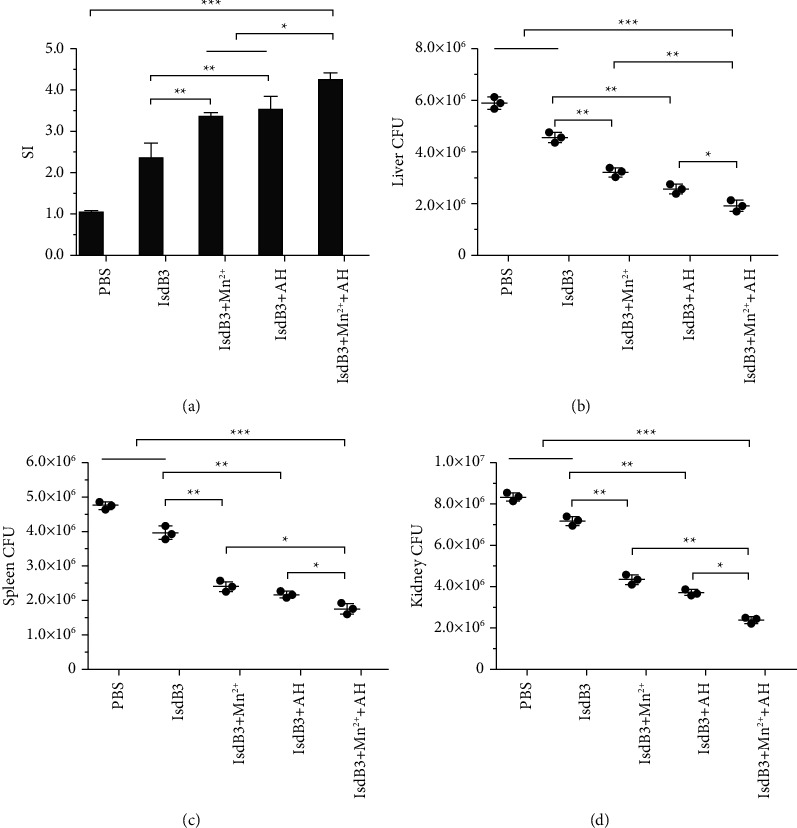
Proliferative responses of spleen lymphocyte and bacterial load in organs from mice. (a) Proliferative response of splenocytes was assessed with a lymphocyte proliferation assay. Seven days after the last immunization, the spleen lymphocytes from the immunized mice (*n* = 3) were obtained, and these cells were treated with IsdB3 proteins for 48 h. A significant difference was observed in the IsdB3 + Mn^2+^ + AH group, in comparison with the IsdB3 group, IsdB3 + Mn^2+^ group, IsdB3 + AH group, and PBS group. (b–d) Bacterial load in organs was analyzed fourteen days after the last immunization. The immunized mice (*n* = 3) were intraperitoneally treated with the dose of 3 × 10^8^ CFU of *S. aureus* strain Newman, and after 48 h, these mice were sacrificed, and their livers (b), spleens (c), and kidneys (d) were obtained, and the grinding fluid of these organs was used to detect the CFU of bacterial colonization. The data were shown as the mean ± SD (*n* = 3). Significant differences were indicated by ^*∗*^*P* < 0.05, ^*∗∗*^*P* < 0.01, and ^*∗∗∗*^*P* < 0.001.

The evaluation of splenic lymphocyte proliferation is shown in Figure 3(a). Statistical analysis showed that the most obvious cell proliferation was exhibited in the IsdB3 + Mn^2+^ + AH adjuvant group, which was much higher than that in the other immunization groups. In addition, there were no differences in the effect of cell proliferation between IsdB3 + Mn^2+^ and IsdB3 + AH adjuvant groups, but cell proliferation of both groups was significantly higher than that of the IsdB3 group and the PBS group. These results indicated that Mn^2+^ and AH adjuvant were able to act synergistically.

### 3.4. Evaluation of Bacterial Load in Organs

The detection analysis of bacterial load is shown in Figures 3(b)–3(d). The number of bacterial loads in the liver, spleen, and kidney from the IsdB3 + Mn^2+^ + AH group was significantly lower than that of the other groups. Compared with the IsdB3 group, the number of bacterial loads in the liver, spleen, and kidney from the IsdB3 + Mn^2+^ group and the IsdB3 + AH group was significantly decreased, and the results indicated that the combination of Mn^2+^ and AH adjuvant obviously increased the immunogenicity of the IsdB3 proteins and effectively protected mice against *S. aureus* infection.

### 3.5. Detection of Specific Antibody Levels

**Figure 4:**
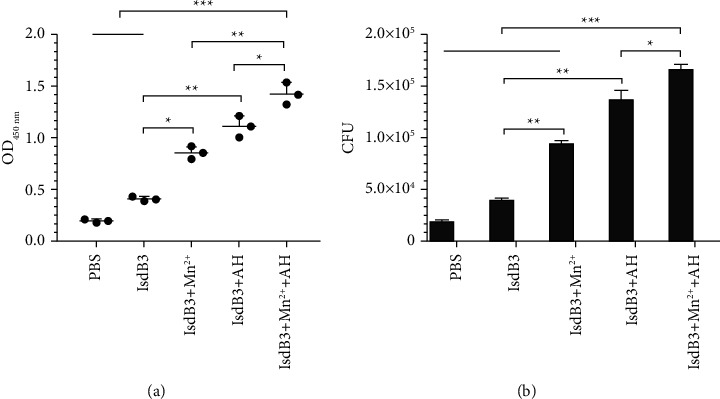
Evaluation of humoral immune response. (a) The detection of antibody levels in mice. The sera in mice were separated and the level of anti-IsdB3 antibodies was analyzed by ELISA fourteen days after the last immunization. (b) The opsonization of the antibody was evaluated by opsonophagocytosis assay. After the sera from immunized mice were incubated with *S. aureus* strain Newman at 37°C for 2 h, this mixture was used to treat peritoneal macrophages from mice at 37°C for 4 h and then the extracellular *S. aureus* Newman strains were removed by gentamicin of 100 *μ*g/mL. Finally, the number (CFU) of bacteria phagocytosed from macrophages was determined in each group. The data were shown as the mean ± SD (*n* = 3). Significant differences were indicated by ^*∗*^*P* < 0.05, ^*∗∗*^*P* < 0.01, and ^*∗∗∗*^*P* < 0.001.

The levels of IgG against IsdB3 were analyzed by indirect ELISA after the serum dilution of 1 : 1000 from immunized mice. As shown in Figure 4(a), the mice in IsdB3 + Mn^2+^ + AH group exhibited the highest levels of specific antibodies (IgG) against IsdB3 among all groups. The level of IgG in the IsdB3 + Mn^2+^ group and the IsdB3 + AH group was also significantly higher than that of the IsdB3 group and the PBS group, which showed that Mn^2+^ and AH adjuvant boosted B cells activation triggered by IsdB3 proteins and enhanced humoral immune response.

### 3.6. Results of Opsonophagocytosis

The opsonization of serum was assessed by opsonophagocytosis assay. As shown in Figure 4(b), the number of the phagocytosed *S. aureus* in the IsdB3 + Mn^2+^ + AH group was the highest among all groups. Moreover, the number of phagocytosed bacteria from the IsdB3 + Mn^2+^ group and the IsdB3 + AH group was significantly higher than that of the IsdB3 group. These results indicated that the combined effect of Mn^2+^ plus AH adjuvant boosted the opsonization of antibodies.

### 3.7. The Survival Rate of Challenge

**Figure 5:**
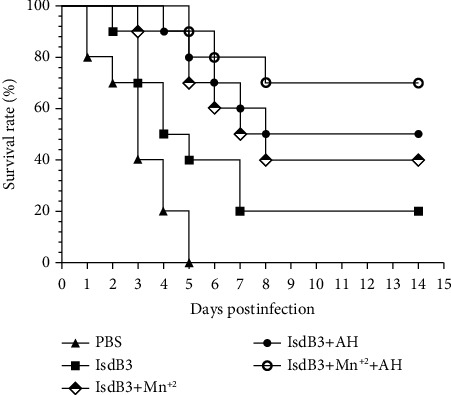
Survival rate of mice in immunized mice. 10 mice in each group were challenged with the lethal doses of 1.5 × 10^9^ CFU of *S. aureus* strain Newman. The survival of mice was confirmed for 14 days after the challenge.

The immunized mice were challenged intraperitoneally with a lethal dose of *S. aureus* strain Newman. The protective efficiency was evaluated by the survival of each group within 14 days of the challenge. As shown in Figure 5, all the mice in the PBS group were dead within 5 days; however, the survival rate of 70% in the IsdB3 + Mn^2+^ + AH group was the highest at 14 days after challenge in all groups. The 14-day survival rate of mice immunized with IsdB3 + AH and IsdB3 + Mn^2+^ was 50% and 40%, respectively, compared with the control groups, including the IsdB3 group and the PBS group, and the survival rate of both groups was significantly higher. Therefore, these results indicated that the combined effect of Mn^2+^ and AH adjuvant was able to increase the immune protective response triggered by IsdB3 proteins in mice.

## 4. Discussion

Here, these data suggested that the combination of Mn^2+^ and AH adjuvant significantly exerted a synergistic effect and obviously enhanced the immunogenicity of IsdB3 proteins. In general, manganese, as a vital trace element, plays a critical role in manipulating metalloenzymes, mainly including Mn superoxide dismutase, pyruvate carboxylase, glutamine synthetase (GS), and arginase [10, 11, 30–32]. In addition, accumulating findings sustained that Mn boosted innate immunity and adaptive immune responses. Mn^2+^ was a potent cGAS activator and elicited the production of IFN-*α*, IFN-*β*, and other cytokines in the absence of infection. Moreover, Mn^2+^ strengthened the host defense against DNA viruses by enhancing the sensitivity of cGAS to double-stranded DNA (dsDNA) [33]. Mn-insufficient mice exhibited significantly enhanced tumor growth and metastasis and markedly decreased tumor-infiltrating CD_8_^+^ T cells. Mn^2+^ greatly promoted DC and macrophage maturation and antigen presentation and augmented CD_8_^+^ T cell and NK cell activation [34]. Importantly, clinical observations indicated that Mn^2+^ administration was able to revive patient's responses to immunotherapy and significantly improved antitumor immunotherapies in various mouse models [34]. More importantly, some recent publications described that manganese could be utilized as an adjuvant to boost immune response in vaccination. A nanodepot of manganese (nanoMn) evokes M1 macrophage polarization and recruits monocytes into inflammatory foci, ameliorates coronavirus-induced tissue damage, and increases the development of virus-specific memory T cells through facilitating antigen presentation [35]. The administration of colloidal manganese salt (Mn jelly (MnJ)) as an adjuvant promoted the production of type I interferon (IFN-I) and the maturation of dendritic cells (DCs) and significantly facilitated the generation of T follicular helper (Tfh) cells, plasma cells (PCs), and RABV-specific antibody-secreting cells (ASCs), following the strengthening of the immunogenicity of rabies vaccines [13]. In addition, MnJ as a mucosal adjuvant induced high levels of secretory IgA. Manganese as an immune potentiator and as a delivery system was able to trigger CD_4_^+^/CD_8_^+^ T cell-mediated immune response [36], thus indicating that manganese will be a promising adjuvant candidate.

In addition, aluminum adjuvants are widely used in preventive vaccines for nearly 100 years. Aluminum adjuvants form a “depot” at the site of injection to release antigen slowly, prolong exposure to antigen-presenting cells, and enhance the expression of major histocompatibility complex (MHC) class II, CD40, CD83, and CD86 molecules on monocytes, and aluminum adjuvants directly induce monocytes to activate T cells by producing proinflammatory cytokines [37]. Moreover, some studies revealed that alum-precipitated proteins elicited a significant increase in the antigen-specific Tfh cells in the draining lymph nodes [38]. At present, aluminum adjuvants have been used to enhance the immune response of hepatitis B surface antigens, human papillomavirus capsid proteins, and inactivated bacterial toxins [39]. However, aluminum adjuvants mainly boost the humoral immune response and have little effect on the Th1-cell-mediated immune response.

In recent years, the combined adjuvants exhibited unique advantages to trigger a desired immune response through evoking a robust mixed Th1/Th2 response. The combination of AH adjuvant with MPLA, CpG, poly (I : C), and MF59 elicited a stronger antibody response and T-cell-mediated immune response [40–44]. In this study, to take full advantage of manganese and aluminum adjuvants, we prepared the combined adjuvants, and the data showed that the combination of Mn2+ plus aluminum adjuvant significantly acted the synergistic immune effect. The administration of IsdB3-Mn^2+^-aluminum adjuvants boosted effectively the proliferation of splenic lymphocytes and the production of IFN-*γ*, IL-4, IL-10, and IL-17 cytokine, significantly enhanced level of the antibodies against IsdB3, and triggered a strong immune response against infection of *S*. *aureus* after challenge. Therefore, the combination of Mn^2+^ and aluminum adjuvant significantly could induce a balanced immune response to reinforce the immunogenicity of IsdB3 proteins. The mechanism underlying the synergistic immune effect induced by Mn^2+^ and aluminum adjuvant may involve the surface of AH attracting Mn^2+^ and antigens, thus allowing for a slow release. Consequently, these components persist, activating a variety of immune cells, including dendritic cells and macrophages. This activation leads to the production of IFN-*γ*, IL-4, IL-10, and IL-17, enhancing the activation of Th1, Th2, and Th17 cells. This, in turn, promotes a heightened humoral immune response and cellular immunity. However, this mechanism needs to be further detected and validated in the future. The state of manganese ions will be changed to further improve the effect of the adjuvant, for example, colloidal state, semisolid states, and nanoparticles. In addition, Mn^2+^ is combined with CpG, poly (I : C), and other adjuvants to form multiple adjuvants, which will further enhance the immunogenicity of IsdB3 proteins. Furthermore, a single antigen as vaccine is difficult to reach a target of effective preventing S. aureus, so IsdB3 proteins in conjunction with other surface proteins of S. aureus, mainly including ClfA, SasA, FnBPA, and GapC, could further boost the immunoprotective effect against *S*. *aureus* with the combinations of Mn^2+^ and aluminum adjuvant, which will be performed in further research. Finally, *S*. *aureus* strain Newman was only performed to challenge assay, and other staphylococcal strains will be used to assess further the immunoprotective effect of Mn^2+^ and aluminum adjuvant.

## 5. Conclusions

Taken together, our data showed that Mn^2+^ with AH adjuvant significantly enhanced the immune response of IsdB_3_ and exhibited a synergistic effect. Therefore, the novel combination of Mn^2+^ and AH adjuvant obviously strengthens the immunogenicity of antigens and will offer a new strategy to increase vaccine efficiency against *S. aureus*.

## Data Availability

The data that support the findings of this study are available from the corresponding author upon reasonable request.

## Abbreviations

Mn: Manganese
AH: Aluminum hydroxide
*S. aureus*: *Staphylococcus aureus*
FDA: Food and Drug Administration
CpG: Cytosine preceding guanosine
IsdB: Iron-regulated surface determinant B
PBS: Phosphate buffer solution
ELISA: Enzyme‐linked immunosorbent assay
PMA: Phorbol myristate acetate
SI: Stimulation index
TSA: Tryptic soy agar
TMB: 3, 3′, 5, 5′-tetramethylbenzidine
CFU: Colony-forming units.
